# A Modified Delphi Consensus on Optimal Pharmacological Management of Insomnia: Insights from a Malaysian Perspective

**DOI:** 10.21315/mjms-07-2025-508

**Published:** 2025-12-31

**Authors:** Ng Chong Guan, Ahmad Hatim Sulaiman, Nor Hazlin Talib, Ahmad Izuanuddin Ismail, Rusdi Abd Rashid, Tan Maw Pin, Thirunavukarasu Rajoo, Tan Chong Tin

**Affiliations:** 1 Department of Psychological Medicine, Faculty of Medicine, Universiti Malaya, Kuala Lumpur, Malaysia; 2 Family Medicine Specialist’s Association, Bandar Baru Bangi, Selangor, Malaysia; 3 Department of Medicine, Faculty of Medicine, Universiti Teknologi MARA, Sungai Buloh Campus, Sungai Buloh, Selangor, Malaysia; 4 Department of Medicine, Faculty of Medicine, Universiti Malaya, Kuala Lumpur, Malaysia; 5 Care Clinics Healthcare Services Sdn Bhd, Kuala Lumpur, Malaysia; 6 Department of Medicine and Neurology Unit, Faculty of Medicine, Universiti Malaya, Kuala Lumpur, Malaysia

**Keywords:** benzodiazepines, cognitive behavioural therapy, consensus, insomnia, lemborexant

## Abstract

Insomnia represents a significant risk factor for impaired daytime functioning and a range of chronic health conditions and has emerged as a growing public health concern in Malaysia. The absence of standardised local guidelines complicates treatment decision-making. This consensus aimed to develop pharmacological treatment recommendations for insomnia in Malaysia using a modified Delphi approach. An eight-member expert panel comprising specialists in sleep medicine, psychiatry, neurology, and pharmacology reviewed high-level evidence on the neurobiology, diagnosis, and treatment of insomnia. A 31-item consensus statement survey addressing the definition, diagnosis, and pharmacological management of insomnia was conducted, with agreement evaluated using a five-point Likert scale. The consensus recommendations were finalised after two Delphi rounds and a final vote, with at least 75% agreement required for each statement. Consensus statements highlighted the need for tailored treatment strategies in Malaysia that combine nonpharmacological and pharmacological interventions. Insomnia was recognised as a chronic condition diagnosed primarily using subjective criteria, with sleep diaries and questionnaires recommended as assessment tools. Benzodiazepines, Z-class drugs, and dual orexin receptor agonists (DORAs) were found to be effective; however, concerns regarding long-term safety and dependency were noted. DORAs demonstrated promise in managing both sleep onset and maintenance, with lemborexant highlighted as a promising pharmacological option. The meeting emphasised the importance of individualised treatment plans and careful risk assessment, particularly for older adults, and provided practical recommendations for the management of insomnia. The integration of Cognitive Behavioural Therapy for Insomnia with pharmacotherapy was strongly advocated. These consensus statements offer a practical foundation for the development of future national guidelines to enhance insomnia care in Malaysia’s primary care settings.

## Introduction

Insomnia is a significant risk factor for a range of adverse health outcomes, including impaired daytime performance, substance misuse, depression, psychiatric disorders, chronic pain, and medical conditions such as obesity, hypertension, cardiovascular disease, and dementia (1). Addressing insomnia requires both clinical interventions at the individual level and broader initiatives to promote sleep health (2). According to the *Diagnostic and Statistical Manual of Mental Disorders*, Fifth Edition (DSM-5), insomnia is characterised by dissatisfaction with sleep quantity or quality that results in impaired daytime functioning (3, 4). Chronic sleep disturbances are highly prevalent in psychiatric conditions, affecting 50% to 80% of adults with mental health disorders (3). The *International Classification of Sleep Disorders*, Third Edition (ICSD-3), further defines chronic insomnia as symptoms occurring at least three times per week for a minimum of three months, accompanied by daytime consequences despite adequate opportunity for sleep (5). Both individual- and organisational-level adjustments can contribute to improvements in sleep outcomes (6).

Approximately 10% of adults experience persistent insomnia, while a further 20% report intermittent symptoms. Women, older adults, and individuals who are socioeconomically disadvantaged are more vulnerable to insomnia, which often persists over time, with a reported 40% continuation rate over five years (2). In Malaysia, sleep-related problems are increasingly recognised as a public health concern. Several studies have examined the prevalence of sleep disturbances in the Malaysian population. A survey of more than 11,000 working adults found that 54.7% reported sleeping fewer than seven hours per night on average, commonly attributed to childcare responsibilities, lifestyle factors, and mental health issues (6). Another study involving over 2,000 primary care patients reported that 60% experienced insomnia symptoms, with 38.9% reporting frequent insomnia, 30.7% meeting criteria for chronic insomnia without daytime consequences, and 28.6% experiencing chronic insomnia with associated daytime dysfunction (7).

Insomnia may precipitate or coexist with a range of mental disorders, with hyperactivation of the stress system, especially the orexinergic pathway, playing a key role (8). As a disorder characterised by hyperarousal, insomnia involves increased activation of both the central (cortical) and peripheral (autonomic) nervous systems (9). Structural alterations have been identified in the amygdala, hippocampus, corpus striatum, and thalamus among individuals with insomnia disorders. In paradoxical insomnia, pronounced shape changes have been observed in the caudate, putamen, and nucleus accumbens, whereas psychophysiological insomnia is characterised by alterations in the thalamus, amygdala, and hippocampus. These distinctions underscore the importance of precise insomnia classification to facilitate more targeted and effective treatment approaches (10). Neuroimaging studies suggest that insomnia may arise from overactivity in corticolimbic regions, reduced gamma-aminobutyric acid (GABA) levels, and altered brain morphometry, with shared physiological signatures evident in electroencephalogram patterns (11, 12).

The optimal management of insomnia remains challenging, with most clinical guidelines recommending Cognitive Behavioural Therapy for Insomnia (CBT-I) as the first-line treatment (13). Nonpharmacological interventions, such as sleep hygiene education and CBT-I, are effective for managing insomnia. Although CBT-I has demonstrated robust efficacy, its implementation faces challenges, including limited availability of trained providers and social stigma (14). Consequently, a more practical approach to nonpharmacological management is required, particularly in primary care settings. Combining CBT-I with pharmacological treatments—such as benzodiazepines (BZDs), BZD receptor agonists (Z-drugs), melatonin, orexin receptor (OXR1/ OXR2) antagonists, sedative antihistamines, and herbal products—can improve sleep outcomes. However, each medication is associated with specific side effects (15). Although approximately one-third of patients achieve complete remission with CBT-I, many continue to experience residual sleep disturbances. Physicians often prescribe United States Food and Drug Administration (FDA)–approved medications tailored to the patient’s particular sleep issues, chronicity, and coexisting conditions (14). At the global level, guidelines from the American College of Physicians (16), the American Academy of Sleep Medicine (AASM) (17), the Veterans Affairs/Department of Defense (18), and European experts (19) highlight the importance of evidence-based recommendations to support healthcare providers in selecting appropriate interventions. However, most pharmacological treatments have been evaluated primarily in placebo-controlled trials, limiting the availability of comparative effectiveness data (20).

Although various international guidelines are grounded in strong evidence, they often provide general recommendations that can be challenging to implement in clinical practice. In Malaysia, there is currently no standardised framework or guideline for the treatment of insomnia, highlighting the need for practical, patient-specific recommendations to support primary care physicians in developing effective management strategies. To address this gap, a consensus meeting was convened to produce a guidance document for clinicians on the pharmacological management of insomnia, employing a modified Delphi approach. The meeting reviewed current clinical practices, evaluated recommendations from established guidelines—including the AASM (2017), VA/DoD (2019), ACP (2016), and European guidelines—and formulated consensus statements and recommendations specifically tailored to the pharmacological management of insomnia in the Malaysian context.

## Methods

### Panel Generation

The expert panel for this Delphi study was established through an iterative and inclusive process, engaging specialists relevant to insomnia care. The panel comprised eight experts representing key disciplines, including sleep medicine (*n* = 1), psychiatry (*n* = 3), neurology (*n* = 2), and pharmacology (*n* = 2). Members were purposively selected based on their clinical expertise, academic credentials, and active involvement in the management of insomnia. The relatively small panel size was intentional to facilitate focused discussion, ensure manageability across iterative rounds, and maintain high-quality expert input. Previous Delphi studies in specialised clinical areas, including insomnia, have demonstrated that smaller panels can achieve valid consensus without compromising methodological rigour (21). Purposive sampling was employed to include experts with established knowledge and experience, consistent with established Delphi methodology and essential for attaining meaningful consensus in specialised areas such as insomnia care (22).

The steering committee ensured representation across multiple specialties to capture diverse perspectives from recognised experts. They identified the following key areas for consideration in the consensus process: the definition of insomnia, its diagnosis, relevant guidelines and recommendations, and the medical management of insomnia.

### Statement Development and Data Collection

A targeted literature review was conducted to gather recent high-level evidence on various topics, including the neuroanatomy, neurophysiology, and pathophysiology of insomnia; its definition and diagnosis; patient screening; nonpharmacological and pharmacological interventions tailored to patient-specific needs, insomnia subtypes, and comorbidities; and individualised pharmacotherapies. The search was performed in PubMed, Embase, and Scopus, covering the period from January 2010 to April 2024, using predefined MeSH terms and keywords related to insomnia. This review was not intended as a full systematic review but as a focused evidence synthesis to support statement development and ensure the inclusion of the most relevant clinical trials, meta-analyses, and international guidelines. Non-English articles, case reports, conference abstracts, and low-quality studies were excluded. This targeted approach ensured that the panel had access to the most clinically pertinent and up-to-date evidence, in accordance with standard Delphi practices. The quality of the supporting evidence was assessed using the Oxford Centre for Evidence-Based Medicine Levels of Evidence (March 2009) (23). This approach does not aim to capture every published study but is consistent with established Delphi methodology, which prioritises supporting expert judgement and facilitating informed consensus rather than conducting an exhaustive systematic review (24). Pre-reading materials were prepared and shared with the panel, followed by an online survey comprising 31 consensus statements.

### Analysis Plan

**Figure 1:**
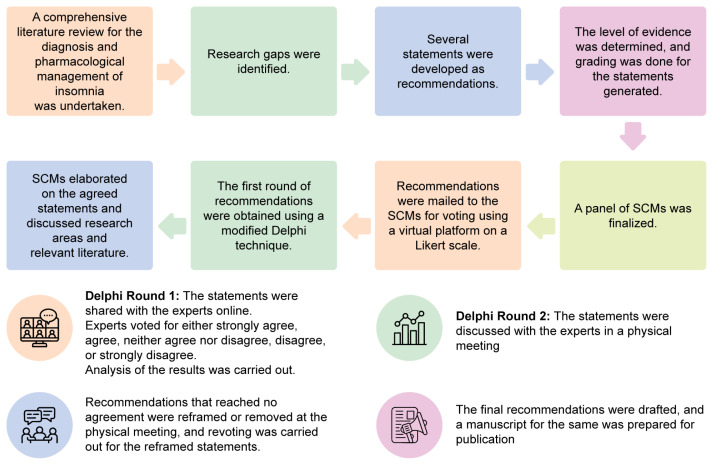
Overview of the consensus development process SCM = steering committee member

The Delphi study survey comprised both quantitative and qualitative components. For each question, responses were collected and the frequencies for all response categories recorded. Responses were measured using a five-point Likert scale: strongly agree, agree, neutral, disagree, and strongly disagree. The co-chairs and working group leaders reviewed the qualitative data submitted via text boxes. Following each survey round, these findings were discussed in a series of meetings to inform decisions on whether statements should be modified, removed, or added. An overview of this process is presented in Figure 1.

**Figure 2:**
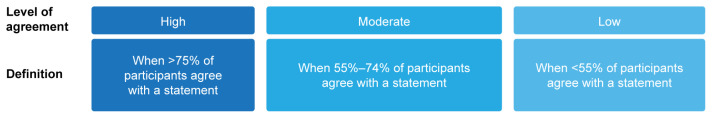
Criteria for determining levels of agreement among the expert panel on the diagnosis and pharmacological management of insomnia

The modified Delphi method was used to gather and analyse expert opinions, resulting in statements classified as having high, moderate, or low agreement. Consensus thresholds were predefined to provide a clear and transparent framework for interpreting expert agreement: ≥ 75% for strong consensus (25), 55% to 74% for moderate consensus, and < 55% for low consensus. These cut-offs align with established Delphi methodology (26), ensuring methodological rigour while maintaining inclusivity. High thresholds indicate robust expert agreement, whereas moderate thresholds highlight areas requiring further discussion, refinement, or validation, thereby strengthening the reliability and clinical relevance of the final consensus. Agreement levels for the diagnosis and management of insomnia are illustrated in Figure 2. Statements achieving moderate or low agreement were extensively discussed, revised, and re-voted during the second round of the modified Delphi process, which was conducted in a physical meeting. A two-round approach was adopted, consistent with evidence indicating that this design achieves stable consensus while minimising participant fatigue and attrition (27). The revised statements were subsequently re-voted to finalise the consensus recommendations. All votes were collected anonymously as part of Delphi process.

## Results

A total of 31 consensus statements were developed, as detailed in Supplementary Table 1. Of these, 21 (68%) statements achieved high agreement, seven (23%) achieved moderate agreement, and three (10%) achieved low agreement.

### Definition of Insomnia

The panel concluded that insomnia in Malaysia should be defined as a chronic condition characterised by persistent difficulty initiating or maintaining sleep despite adequate opportunity and conducive sleep conditions, resulting in significant impairment of daytime functioning (Statement I2). They agreed that the recommended sleep duration for Malaysian adults should ideally be ≥ 7 hours, while emphasising the need for individualisation based on lifestyle and daytime functioning (Statement I1). The discussion further clarified that insomnia is a distinct clinical entity requiring prompt medical attention due to its severity. Although the term “distinct” was debated—some panellists suggesting it could imply an absence of underlying causes—consensus was reached to frame the statement as: “Insomnia represents a distinct clinical entity that requires immediate clinical attention” (Statement I3). Additionally, the panel supported the statistic that 10% to 30% of adults in Malaysia experience chronic insomnia, affirming its relevance to clinical practice (Statement I4).

### Diagnosis of Insomnia

Measuring treatment success in insomnia remains challenging, as objective definitions of good sleep may not always align with patients’ experiences. This reliance on subjective assessments has raised concerns regarding the robustness of the diagnosis, as reflected by a low agreement level of 50% for this statement (Statement D1). Diagnostic tools such as the Consensus Sleep Diary were supported, with polysomnography (PSG) recommended for selective use. Experts highlighted that PSG data often correlate poorly with subjective sleep assessments, indicating that while PSG can be informative, it should not serve as the sole basis for diagnosis (Statement D2). The panel emphasised that validated questionnaires—such as the Insomnia Severity Index (ISI), Sleep Condition Indicator, and Epworth Sleepiness Scale—are essential for assessing insomnia severity and its impact on daytime functioning (Statement D3). They also recommended the removal of the statement regarding dysfunctional beliefs and attitudes about sleep (DBAS), as it was deemed not relevant in the Malaysian context (Statement D4).

### Treatment for Insomnia

Experts highlighted the importance of carefully weighing the benefits and drawbacks when determining the duration of insomnia treatment, with 85% agreement on this statement (Statement G1). They advocated for individualised pharmacological interventions, considering both physical and psychiatric risks (e.g., depression or anxiety) as well as lifestyle factors (Statement P2). While medications such as BZDs, lemborexant, and suvorexant are effective for acute insomnia, experts cautioned against long-term use due to concerns regarding tolerability and limited safety data (Statements P3 and P10). BZDs should be avoided in elderly patients, particularly those with dementia or cognitive impairment, owing to their heightened sensitivity (Statement P1). Furthermore, 75% of experts agreed that zopiclone is effective in treating insomnia by reducing sleep latency, nocturnal awakenings, and wake-after-sleep onset while increasing total sleep time. However, they emphasised that the dose should be gradually tapered to minimise the risk of rebound insomnia (Statement P5).

The consensus regarding dual orexin receptor antagonists (DORAs), such as lemborexant, indicates favourable long-term safety and tolerability, with 75% agreement that these agents are associated with minimal treatment-emergent adverse events and no next-morning sleepiness or withdrawal symptoms (Statement G4). Zolpidem is effective for sleep initiation due to its rapid onset and is preferred over BZDs for short-term use; however, concerns remain regarding next-day cognitive impairment in older adults (Statement P4). Experts recommend prescribing the minimum effective dose of Z-class drugs for less than two weeks, particularly in individuals over 60 years of age, in conjunction with nonpharmacological interventions (Statement P6). Although Z-class drugs, such as zolpidem, demonstrate greater efficacy than placebo in improving sleep parameters, they carry an increased risk of addiction (Statement P7).

The panel also noted that herbal medicines such as valerian, chamomile, and kava, although commonly used for insomnia, demonstrate no significant efficacy compared with placebo and are discouraged by current guidelines, with 62.5% of the panel in agreement (Statement G2). Furthermore, 75% of experts highlighted that mirtazapine is frequently prescribed for insomnia, despite not being specifically addressed in existing guidelines. They emphasised the need for caution in patients with metabolic disorders due to the risk of weight gain (Statement G3). Additionally, only 25% of experts agreed that quetiapine is occasionally used off-label for insomnia, whereas 62.5% disagreed, citing concerns regarding its addiction potential (Statement G7).

### Positioning of DORAs

Lemborexant was recommended for sleep maintenance in patients with insomnia, including those transitioning from BZDs, with 87.5% agreement on its effectiveness in improving sleep parameters (Statements G5, G6, and P9). Transitioning to lemborexant has demonstrated high effectiveness, with 62.5% agreement regarding improved patient satisfaction and tolerability (Statement P8). Lemborexant is recognised for its superior efficacy compared with placebo (87.5% agreement) and its low potential for dependence, coupled with minimal side effects, supporting its real-world effectiveness (Statements P9 and P11). It is effective across all age groups and maintains efficacy over a 12-month period, with unanimous agreement (100%) (Statement P12). Furthermore, its efficacy in maintaining sleep appears to be dose-dependent, with 87.5% agreement (Statement P13). Experts agreed that no single drug should be recommended as first-line treatment for primary insomnia. Pharmacological therapy should instead be combined with nonpharmacological interventions, considering individual patient factors (Statement P14). Lemborexant’s sustained effectiveness over 12 months highlights its long-term benefits for adults with insomnia, with 75% agreement among panellists (Statement P15). Additionally, it minimises next-day impairment while maintaining a favourable efficacy and safety profile (Statement P16). Revised statements are provided in Supplementary Table 2.

## Discussion

### Definition and Diagnosis of Insomnia

Challenges in applying objective definitions of insomnia arise because total sleep requirements vary according to daily activities and age. The panel noted that defining insomnia and measuring treatment success are complex due to individual differences in sleep needs. Chronic insomnia is clinically diagnosed in accordance with DSM-5, ICSD-3, and International Classification of Diseases-11 criteria, encompassing difficulties with sleep onset, maintenance, early awakening, and daytime impairment despite adequate opportunity for sleep (19). Experts expressed diverse views on insomnia diagnosis. The panel emphasised the importance of subjective measures, such as feeling refreshed upon waking and experiencing minimal daytime sleepiness, as key indicators of satisfactory sleep. While this approach prioritises patient-reported experiences, the consensus acknowledges the limitation of not incorporating biological or objectively measurable parameters.

The panel indicated that PSG should be reserved for specific cases and that PSG results should be interpreted with caution, given that the test is conducted in a controlled laboratory environment. This emphasises the need for a nuanced approach that considers both subjective reports and objective measurements in select circumstances. Experts advocated the use of the Consensus Sleep Diary to capture the day-to-day variability of insomnia during treatment and for long-term reassessment to monitor progress and potential relapses. They also suggested incorporating sleepiness scales used in driving licence examinations alongside standard sleep indices in insomnia assessments. Although the ISI has been proposed as a diagnostic tool, concerns remain regarding its adaptation for diagnosis, as it primarily measures sleepiness. However, neighbouring countries such as Indonesia and Thailand have endorsed the ISI as a screening tool within their insomnia guidelines, providing a regional framework that could inform its use in Malaysia. This multifaceted approach underscores the importance of tailored assessment for the diagnosis and management of insomnia.

### Nonpharmacological Interventions

A recurring theme in the panel discussions was the importance of prioritising nonpharmacological interventions as the first-line of treatment. These include behavioural therapies, CBT-I, and lifestyle modifications aimed at improving sleep hygiene. Although patients in high-stress environments or those who travel frequently may request pharmacotherapy, nonpharmacological interventions remain the recommended first-line approach. Primary care providers should be equipped to counsel patients on the importance of circadian rhythm regulation, as well as the risks and benefits of pharmacotherapy, especially in Malaysia, where the availability of pharmacological treatments is limited. Classifying patient demographics is essential for early intervention, allowing for tailored treatment strategies. Screening can help identify individuals in the early stages of insomnia, where nonpharmacological interventions are typically employed first. Pharmacological therapy should only be initiated if nonpharmacological interventions fail to manage the condition adequately.

### Pharmacological Interventions

Current recommendations advise limiting pharmacological treatment for insomnia to four weeks due to the risk of rapid tolerance development. Experts debated the optimal duration, with some advocating two weeks to minimise the risk of dependence, while others suggested five days to achieve faster therapeutic effects. Consensus was reached on individualising treatment duration according to patient-specific factors, with 90% agreement on the importance of personalised care.

#### BZDs

Commonly prescribed BZDs for insomnia include triazolam, temazepam, estazolam, quazepam, and flurazepam, all classified as Schedule IV controlled substances (5). Despite their known risks, BZD hypnotics remain widely prescribed for insomnia (13). A clear treatment strategy is essential to avoid long-term BZD use, implement alternative therapies and manage discontinuation effectively. In a recent Malaysian study, 12.16% of patients attending psychiatry outpatient clinics were prescribed BZDs (28). A systematic review confirmed the short-term efficacy of BZDs over placebo and alternatives such as melatonin and ramelteon. However, it also highlighted significant drawbacks, including adverse effects and higher dropout rates due to adverse drug reactions (ADRs). Limited long-term safety data make BZDs less suitable for extended treatment, despite their demonstrated effectiveness (20).

Experts recommended the selective use of BZDs based on individual patient factors and comorbidities, primarily for short-term management of insomnia when nonpharmacological interventions are ineffective. Long-term use is discouraged due to the risks of dependence, cognitive impairment, and next-day residual effects. The panel unanimously agreed that BZDs should be avoided in patients at increased risk of falls or fractures and emphasised the importance of careful monitoring. BZDs should not be considered first-line treatments in Malaysia-specific guidelines, with safer alternatives, including non-BZD hypnotics (Z-class drugs) and newer agents such as lemborexant, preferred for long-term management.

#### BZD Receptor Agonists (Z-drugs)

Z-drugs, including zolpidem, eszopiclone, and zaleplon, are commonly used for insomnia and are classified as Schedule IV controlled substances (5). These drugs are listed in the Beers Criteria due to their increased risk of ADRs in elderly patients and should not be used for more than three months. Prolonged use beyond six months is associated with withdrawal symptoms in nearly one-third of patients (28). During the consensus meeting, experts emphasised the importance of individualising treatment based on patients’ social demographics and clinical backgrounds, noting that Z-drugs are generally preferred over BZDs in older patients. Overall, the panel highlighted the need for cautious prescribing and careful monitoring when using Z-drugs in clinical practice.

#### DORAs

DORAs block OX1R and OX2R, providing effective treatment for insomnia when appropriately dosed (5). The FDA-approved agents, suvorexant (2014), lemborexant (2019), and daridorexant (2022), are effective for both sleep onset and maintenance. However, suvorexant and daridorexant are currently unavailable in Malaysia. Lemborexant, available since 2022, is considered safe, with no evidence of cognitive dysfunction, increased risk, or dependence over at least one year. A recent review indicates that DORAs, including lemborexant and seltorexant, demonstrate favourable efficacy, although concerns remain regarding long-term safety and the availability of extended data (20).

#### Lemborexant: Efficacy and Safety

In a phase 3 study, lemborexant (5 mg and 10 mg) significantly improved subjective sleep onset and maintenance compared with placebo over six months, reducing latency to persistent sleep, enhancing sleep efficiency, and decreasing wake-after-sleep onset. Lemborexant also outperformed zolpidem in reducing wake-after-sleep onset during the second half of the night (29). The treatment was generally well-tolerated, with most adverse effects being mild or moderate (30). Experts expressed strong support for the use of DORAs, particularly lemborexant, as a first-line pharmacological option for insomnia following the failure of nonpharmacological interventions. They highlighted that DORAs offer an innovative mechanism of action by targeting the orexin system, which plays a crucial role in regulating wakefulness.

Lemborexant demonstrates superior efficacy compared with BZD receptor agonists in both sleep onset and maintenance, showing better subjective sleep latency than suvorexant and representing a strong option for patients transitioning from BZDs (30). A retrospective analysis reported that 66.7% of patients successfully transitioned from BZDs to lemborexant, with predictors of success including lower initial BZD doses and ongoing dose reduction. Lemborexant effectively manages insomnia, reduces dependence, and improves overall sleep outcomes (31). A post hoc analysis showed that lemborexant 10 mg produced greater improvements in sleep onset and total sleep time in patients with a history of depression compared with lemborexant 5 mg and placebo (32). Lemborexant is approved in Japan and Singapore, and global studies indicate no rebound insomnia or withdrawal effects after 12 months of use (33). A review of nine clinical studies found minimal next-day functional impairment, no significant postural or cognitive decline, and reported mild to moderate somnolence as the most common adverse event (34).

Experts emphasised that lemborexant has a favourable safety profile, with minimal potential for dependence and few side effects. It demonstrates efficacy across all age groups and maintains effectiveness over 12 months without an increase in adverse events. Lemborexant is particularly effective in a dose-dependent manner for sleep maintenance and has minimal impact on next-day functioning, supporting improved patient outcomes. While its long-term benefits are well established, experts recommend combining pharmacological and nonpharmacological approaches, tailored to individual patient needs, to achieve optimal insomnia management.

#### Melatonin Receptor Agonists (Ramelteon)

The melatonergic system represents a potential target for the treatment of insomnia and circadian rhythm disturbances. Although exogenous melatonin has demonstrated some benefits in specific populations, such as older adults and individuals with neurodevelopmental disorders, including attention deficit hyperactivity disorder, robust evidence supporting its clinical efficacy for insomnia remains limited (26). Recent systematic reviews report mixed findings regarding melatonin’s effectiveness and safety profile, and its use for insomnia remains off-label, despite some evidence suggesting potential benefits for various sleep disorders in adults. Melatonin is not widely available in Malaysia and is not approved as an over-the-counter (OTC) supplement by the Ministry of Health, as it is classified as a hormone. There is a need for further high-quality empirical research to evaluate the effectiveness and safety of melatonin in the Malaysian context (35).

#### Antidepressants

Experts recommended including mirtazapine in insomnia treatment guidelines to support informed clinical decision-making. Although mirtazapine is frequently used in clinical practice for insomnia, current guidelines do not specifically address its use for this condition. The panel highlighted that, due to its side-effect profile—particularly the potential for weight gain—clinicians should exercise caution when prescribing mirtazapine to patients with metabolic disorders. This consideration is crucial, as it affects both treatment efficacy and the overall health of these patients.

#### Antipsychotics

Experts discussed that quetiapine, an antipsychotic, is occasionally used off-label as an adjunct therapy for insomnia, particularly in patients with coexisting psychiatric conditions such as schizophrenia. While some panel members initially cited “significant addiction potential,” others argued that quetiapine is more commonly associated with metabolic disturbances, next-day sedation, and daytime somnolence. Consequently, the statement was revised to refer to “clinical side effects” rather than addiction. However, the consensus strength for this statement was low, highlighting the need for further discussion and consideration of evidence regarding the safety profile and potential risks of quetiapine’s off-label use in insomnia management.

#### OTC Products

OTC products for insomnia include melatonin, diphenhydramine, doxylamine, L-tryptophan, valerian, and chamomile. All, except diphenhydramine and doxylamine, are classified as dietary supplements (5). Experts discussed the efficacy of herbal medicines commonly used for insomnia, such as valerian, chamomile, and kava. Despite their widespread use, studies show no statistically significant benefit compared with placebo, leading experts to discourage their use. It was also noted that certain herbal teas may contain stimulants, which could counteract their intended calming effects. Furthermore, these remedies are not highly relevant in the Malaysian context, supporting their exclusion from clinical recommendations.

**Figure 3:**
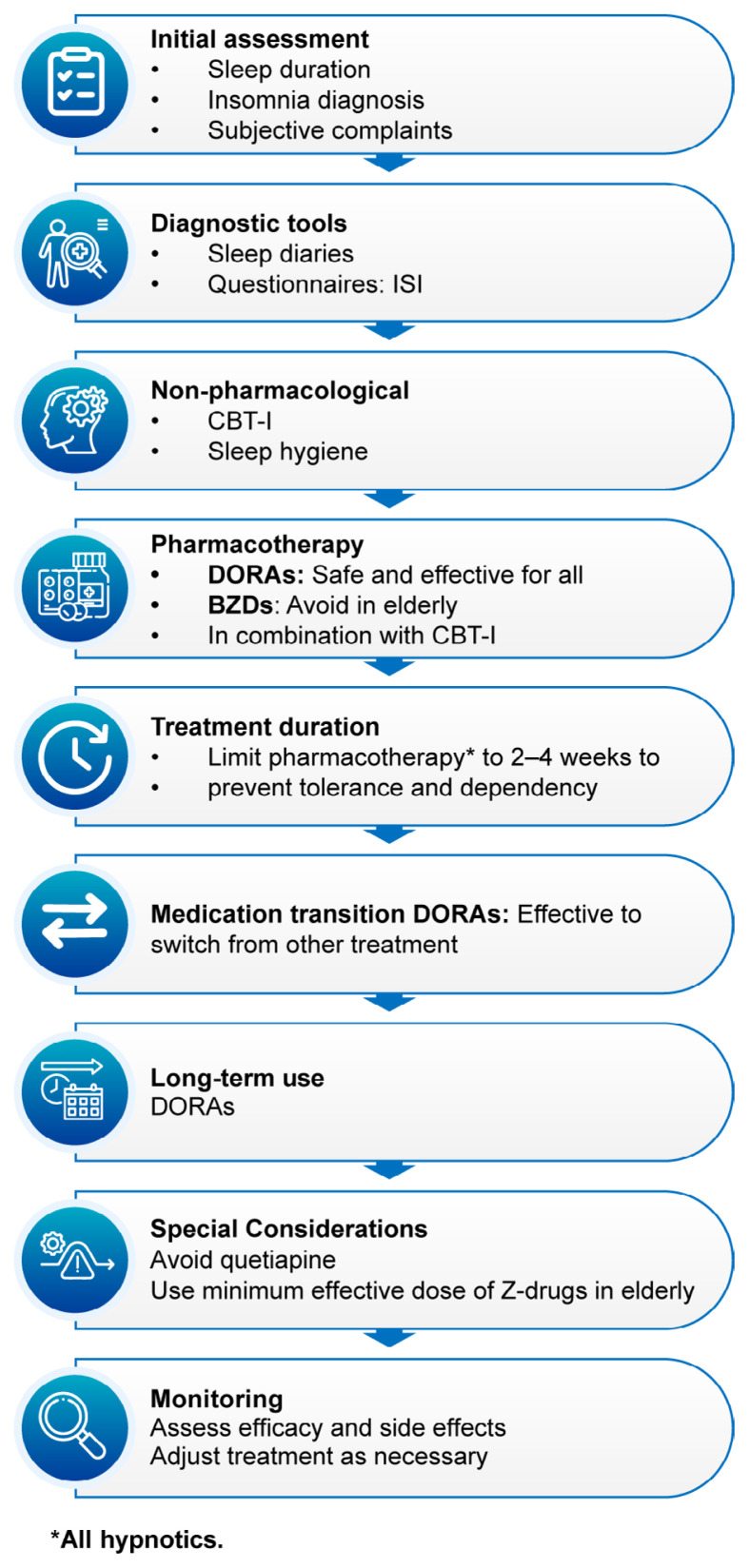
Overview of the clinical pathway from diagnosis to treatment selection and monitoring in patients with insomnia BZD = benzodiazepine; CBT-I = Cognitive Behavioural Therapy for Insomnia; DORA = dual orexin receptor antagonist; ISI = Insomnia Severity Index; Z-Drugs = nonbenzodiazepines

Experts emphasised the importance of tailoring insomnia treatment to individual patient factors, particularly considering coexisting medical or psychiatric conditions. Figure 3 provides an overview of the process from diagnosis to ongoing monitoring in patients with insomnia, as discussed by the expert panel.

### Proposed Algorithm for the Management of Insomnia in Malaysia

In Malaysia, diagnosing insomnia involves assessing symptoms such as difficulty initiating or maintaining sleep, early awakening, and daytime dysfunction, including fatigue and mood disturbances. The ICSD-3 categorises insomnia into chronic, short-term, and other specified disorders. Experts define insomnia as a chronic condition that significantly impairs daytime functioning and emphasise the importance of obtaining a thorough clinical history. Diagnosis relies on ICSD-3 criteria, requiring confirmation of at least one core symptom while excluding other potential disorders. PSG should be used selectively to rule out comorbidities, as its findings often do not align with subjective reports. Self-reported instruments, such as the Pittsburgh Sleep Quality Index and ISI, are crucial for evaluating severity and monitoring treatment progress. The panel supports the use of culturally appropriate assessment tools and highlights the importance of individualised treatment plans, ideally targeting a minimum of seven hours of sleep, with ongoing evaluation to ensure treatment efficacy and manage potential relapses.

Treatment of insomnia involves both behavioural and pharmacological interventions, with CBT-I recommended as the first-line approach. Despite its proven efficacy, implementation is often limited by a shortage of trained therapists, making pharmacotherapy more commonly used in practice. Nonpharmacological interventions, such as sleep hygiene education and lifestyle modifications, are essential and should be prioritised, particularly for patients in high-stress environments. Combination therapy, integrating CBT-I with pharmacotherapy, has been shown to yield better outcomes than pharmacotherapy alone. Primary care physicians play a crucial role in delivering nonpharmacological treatments and educating patients about circadian rhythms, as well as the risks and benefits of pharmacotherapy. Early screening is important to identify patients in the initial stages of insomnia, enabling tailored treatment strategies that emphasise nonpharmacological approaches first. Pharmacotherapy should only be considered when nonpharmacological interventions fail to achieve satisfactory results.

Experts emphasised that the selection of pharmacological treatments for insomnia should take into account factors such as symptom severity, duration, drug efficacy and safety, and the presence of comorbidities. Medications were classified into several categories: BZDs, Z-drugs, DORAs, melatonin receptor agonists, antidepressants, antipsychotics, and OTC products. Caution was advised regarding the long-term use of BZDs and Z-drugs due to the risks of cognitive dysfunction, dependence, and tolerance. The panel recommended limiting pharmacological treatment to four weeks, although some experts advocated a two-week maximum to minimise the risk of dependency. Strong emphasis was placed on individualising treatment according to each patient’s physical and psychiatric comorbidities. Experts recommended selective use of BZDs for short-term management and discouraged their use in elderly patients due to the increased risk of falls and fractures.

DORAs, particularly lemborexant, were preferred as first-line pharmacological treatments following the failure of nonpharmacological interventions, owing to their favourable efficacy and safety profile. Experts highlighted their minimal adverse effects and low potential for dependence, making them suitable for managing chronic insomnia. However, the panel stressed that no single drug should be universally recommended; treatment must be individualised to each patient’s needs. While melatonin is not widely approved in Malaysia, experts acknowledged some evidence supporting its potential role in treating insomnia, though they emphasised the need for further research. The inclusion of mirtazapine in treatment guidelines was recommended, given its frequent use in clinical practice, with caution advised due to the risk of weight gain. The off-label use of quetiapine for insomnia was also discussed, with concerns raised regarding metabolic side effects and next-day sedation. Experts evaluated OTC products, including melatonin, diphenhydramine, and herbal remedies such as valerian, noting variability in their efficacy and safety profiles. Overall, the consensus underscored the importance of personalised treatment strategies, careful monitoring, and further research into the long-term effects of pharmacological interventions for insomnia.

**Figure 4:**
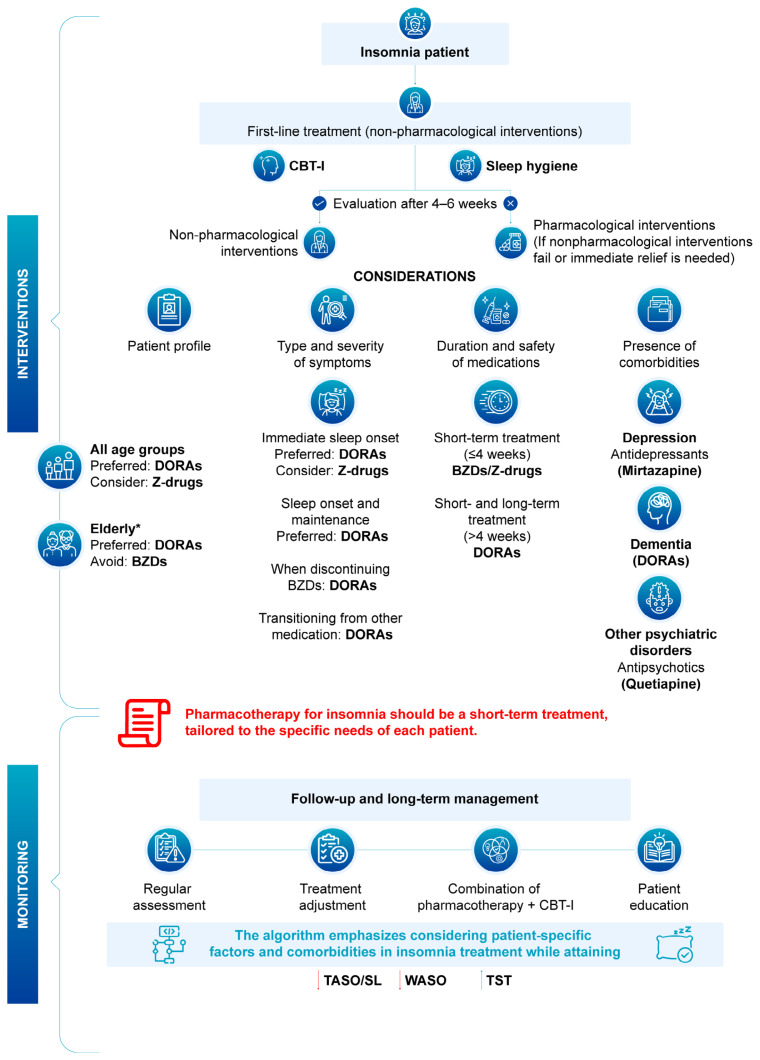
Algorithm for the management and monitoring of insomnia treatment in Malaysia BZD = benzodiazepine; CBT-I = Cognitive behavioural therapy for insomnia; DORA = dual orexin receptor antagonist; SL = sleep latency; TASO = time after sleep onset; TST = total sleep time; WASO = wake-after-sleep onset; Z-Drugs = nonbenzodiazepines; *Patients with dementia, cognitive impairment, or those prone to falls and fractures Drugs discussed during Delphi consensus—BZDs: No specific names were discussed; Z-drug: Zolpidem; DORA: Lemborexant

However, this consensus is subject to several potential limitations. Individual statements evolved throughout the process, reflecting changes as new issues were considered and the diverse opinions of experts were incorporated. Study limitations include the small number of experts, their distribution across specialties, clinic types, and regions, as well as potential self-selection bias arising from purposive sampling. Key stakeholders, including patients, nurses, and policymakers, were not involved in the process. Figure 4 illustrates the proposed pharmacotherapy approach, tailored to different patient populations and characteristics.

## Conclusion

This represents the first consensus report on insomnia management specifically developed for the Malaysian population, addressing a significant gap in local guidelines. The consensus was particularly important given the rising prevalence of insomnia in Malaysia, which adversely affects daily functioning and is associated with serious health conditions, including depression, obesity, and cardiovascular disease. The key takeaways from this study are the following:

- Nonpharmacological interventions, such as CBT-I and lifestyle modifications, should be prioritised before initiating pharmacotherapy.
- Pharmacological treatments, including BZDs, Z-drugs, neurohormones, orexin receptor antagonists, and herbal products, demonstrate varying levels of efficacy and safety.
- BZDs should be restricted to short-term use due to risks such as cognitive dysfunction and dependence, whereas Z-drugs are generally preferred for their better tolerability.
- DORAs may be considered in Malaysia for sleep-maintenance insomnia, given their strong efficacy and safety profiles, minimal potential for dependence, and limited cognitive effects.
- DORAs provide a well-tolerated alternative to BZDs, providing long-term benefits without rebound insomnia or withdrawal symptoms.
- Treatment plans should be individualised, taking into account factors such as age, lifestyle, and comorbidities, and ideally combining nonpharmacological and pharmacological approaches.

In conclusion, the experts emphasised the importance of a comprehensive, patient-specific approach to insomnia management, balancing pharmacological and nonpharmacological interventions. While international guidelines provide a useful framework, Malaysia’s healthcare system would benefit from locally tailored guidelines. Such guidelines should incorporate practical tools, such as the ISI for screening, promote sleep hygiene and nonpharmacological interventions, and carefully consider the safety, efficacy, and individualisation of pharmacotherapies, including agents such as lemborexant.

## Supplementary Data

**Supplementary Table 1:** Consensus statements and their levels of agreement among the expert panel

No.	Consensus statement	Agree	Neutral	Disagree	Level of agreement *
**Definition of insomnia**					
I1	The sleep duration should be ≥ 7 hours for Malaysian adults to avoid issues related to sleep insufficiency.	75%	12.5%	12.5%	High
I2	Insomnia disorder is defined as a chronic condition characterised by persistent difficulty in initiating or maintaining sleep despite having adequate opportunity and conducive conditions for sleep, and it is associated with significant impairment in daytime functioning.	100%	-	-	High
I3	Insomnia disorder represents a distinct clinical entity, no longer classified as solely primary or secondary, but recognised as often coexisting with various medical and psychiatric conditions, particularly depression, which commonly accompanies it in clinical practice.	62.5%	25%	12.5%	Moderate
I4	Recent statistics from an online, self-administered, face-validated questionnaire (Zahari et al., 2022) indicate that 10% to 30% of adults have chronic insomnia. Do you agree with this finding?	100%	-	-	High
**Diagnosis of insomnia**					
D1	The diagnostic criteria for insomnia are based solely on subjective complaints of poor sleep quantity and/ or quality, without including any biological or other objectively measurable parameters.	50%	12.5%	37.5%	Low
D2	The diagnosis of insomnia using PSG should be reserved for specific cases, as PSG data do not strongly correlate with subjective assessments of sleep.	62.5%	25%	12.5%	Moderate
D3	The Consensus Sleep Diary should be used for at least one to two weeks to assess the day-to-day variability of insomnia. Validated questionnaires (ISI, SCI, and ESS) should be used to evaluate insomnia severity and its daytime impact before and during treatment, as well as for long-term re-evaluation or relapse monitoring.	87.5%	-	12.5%	High
D4	The DBAS is recommended for assessing negative behaviours and cognitive processes that perpetuate insomnia. PSG is suggested for suspected sleep-related breathing or movement disorders, while actigraphy may aid in diagnosing circadian rhythm disturbances associated with insomnia.	75%	25%	-	High
**Guidelines recommendations for insomnia**					
G1	According to current evidence, it is recommended to limit drug treatments for insomnia to four weeks due to the rapid development of tolerance. Avoiding dose increases is advised to prevent dependency. Extended treatment periods may be considered in certain cases, with careful consideration of the benefits and drawbacks.	87.5%	12.5%	-	High
G2	While herbal medicines such as valerian, chamomile, and kava are among the most commonly used CAMs for insomnia, there is no statistically significant difference in their efficacy compared with a placebo, and their use is discouraged by guidelines.	62.5%	37.5%	-	Moderate
G3	While mirtazapine is commonly used in clinical practice for patients with insomnia, none of the guidelines address its use. Moreover, caution should be exercised in patients with metabolic disorders due to the risk of weight gain.	75%	25%	-	High
G4	DORAs show excellent long-term safety and tolerability in patients with insomnia. They maintain a consistent safety profile with minimal TEAEs and do not cause next-morning sleepiness or withdrawal symptoms after discontinuation.	75%	25%	-	High
G5	LEM is recommended as a first-line treatment for sleep maintenance insomnia when initiating treatment.	75%	25%	-	High
G6	LEM is recommended as a treatment for sleep-maintenance insomnia during BZD discontinuation.	75%	12.5%	12.5%	High
G7	Quetiapine, an antipsychotic, is occasionally used off-label as an adjunct therapy for insomnia in patients with other psychiatric conditions (e.g., schizophrenia) but is associated with a significant risk of dependence.	25%	12.5%	62.5%	Low
**Pharmacotherapies for insomnia**					
P1	BZDs should be used with caution in elderly patients with dementia, cognitive impairment, or those prone to falls and fractures, due to their heightened sensitivity to depressants affecting the central nervous system.	100%			High
P2	Zolpidem can be a suitable treatment for insomnia, but prescribing and dosing should be determined on a case-by-case basis, considering the patient’s physical and psychiatric risk factors.	100%			High
P3	Many licensed drugs, such as BZDs and suvorexant, are effective for the acute treatment of insomnia. However, they often have poor tolerability, and data on their long-term effects are limited.	62.5%	25%	12.5%	Moderate
P4	Both zolpidem and zopiclone effectively initiate sleep with a rapid onset. Zolpidem, preferred for short-term use, raises concerns about next-day cognitive impairment in older adults. It is as effective as zopiclone, with fewer rebound effects upon discontinuation and better overall tolerability.	87.5%	12.5%	-	High
P5	Zopiclone effectively treats insomnia by reducing SL, nocturnal awakenings, and WASO, while increasing TST. However, its dose should be gradually tapered to minimise the risk of rebound insomnia.	75%	25%	-	High
P6	The minimum effective dose of BZDs and Z-class drugs should be used only when essential and for less than four weeks, especially in individuals over 60 years of age.	100%	-	-	High
P7	Z-class drugs, particularly zolpidem, are more effective than placebo in improving sleep parameters such as SL, WASO, TST, and SE, but they are associated with a higher risk of dependence.	87.5%	12.5%	-	High
P8	Transitioning to LEM from other insomnia medications is highly effective, with 95% of patients successfully switching by the end of titration. Patients reported improved satisfaction based on PGI-I and ISI scores, with mostly mild or moderate TEAEs. LEM provides a well-tolerated and beneficial option for patients dissatisfied with current treatments, potentially reducing or discontinuing BZRA use.	62.5%	37.5%	-	Moderate
P9	DORAs demonstrate superior efficacy compared with placebo in addressing both sleep-onset and sleep-maintenance insomnia. Specifically, LEM (10 mg) achieves the most significant reduction in WASO within minutes.	87.5%	12.5%	-	High
P10	GABAergic drugs, such as BZDs and Z-class drugs, have been the mainstay of insomnia treatment, but their short-term benefits should be weighed against long-term risks, including cognitive impairment, falls, fractures, tolerance, and dependence.	87.5%	12.5%	-	High
P11	LEM exhibits a low potential for dependence, minimal muscle relaxant effects, limited somnolence, and little impact on cognitive function, suggesting it may be a safe and effective option for treating insomnia in real-world clinical practice.	100%			High
P12	LEM is effective in both older and younger adults without an increased risk of AEs. It maintains efficacy and safety over 12 months, with no reduction in effectiveness, withdrawal symptoms, or rebound insomnia.	100%	-	-	High
P13	LEM is more effective in maintaining sleep in a dose-dependent manner, with the 5 mg dose (LEM5) being well tolerated.	87.5%	12.5%	-	High
P14	LEM’s efficacy in key sleep parameters (TST, LPS, and SE), along with its safety profile—comparable to other treatments in terms of SAEs and withdrawals due to AEs—makes it a compelling first-line treatment option for primary insomnia. Its consistent safety and effectiveness across all age groups further support its widespread clinical use.	50%	37.5%	12.5%	Low
P15	LEM demonstrates sustained effectiveness in improving sleep onset and maintenance over twelve months of continuous treatment, with no evidence of rebound insomnia or withdrawal symptoms upon discontinuation, indicating potential long-term benefits for adults with insomnia.	75%	25%	-	High
P16	LEM shows minimal impairment of next-day functioning in people with insomnia, with no significant differences from placebo in postural stability, driving performance, or most cognitive measures.	62.5%	37.5%	-	Moderate

* As stated in Figure 2;

AE = adverse event; BZD = benzodiazepine; BZRA = benzodiazepine receptor agonist; CAM = complementary and alternative medicine; DBAS = dysfunctional beliefs and attitudes about sleep; DORA = dual orexin receptor antagonist; ESS = Epworth Sleepiness Scale; GABA = gamma-aminobutyric acid; ISI = Insomnia Severity Index; LEM = lemborexant; LEM5 = lemborexant 5 mg; LPS = latency to persistent sleep; PGI-I = Patient Global Impression – Insomnia; PSG = polysomnography; SAE = serious adverse event; SCI = Sleep Condition Indicator; SE = sleep efficiency; SL = sleep latency; TEAE = treatment-emergent adverse event; TST = total sleep time; WASO = wake-after-sleep onset

**Supplementary Table 2:** Revised consensus statements

No.	Consensus statement	Statement revisions
1	The sleep duration should be ≥ 7 hours for Malaysian adults to avoid issues related to sleep insufficiency.	Sleep duration should be ≥ 7 hours among Malaysian adults to avoid issues related to sleep insufficiency, considering patient-specific factors such as lifestyle and level of daytime functioning.
2	Insomnia disorder represents a distinct clinical entity, no longer classified as solely primary or secondary, but recognised as often coexisting with various medical and psychiatric conditions, particularly depression, which commonly accompanies it in clinical practice.	Insomnia represents a distinct clinical entity that requires immediate clinical attention.
3	The diagnostic criteria for insomnia are based solely on subjective complaints of poor sleep quantity and/ or quality, without including any biological or other objectively measurable parameters.	Medical attention for insomnia can be based on subjective complaints of poor sleep quantity and/ or quality.
4	The Consensus Sleep Diary should be used for at least one to two weeks to assess the day-to-day variability of insomnia. Validated questionnaires (ISI, SCI, and ESS) should be used to evaluate insomnia severity and its daytime impact before and during treatment, as well as for long-term re-evaluation or relapse monitoring.	A sleep diary can be used as a clinical tool to assess the day-to-day variability of insomnia. In contrast, the ISI can be used for diagnostic screening to evaluate insomnia severity and its daytime impact.
5	The DBAS is recommended for assessing negative behaviours and cognitive processes that perpetuate insomnia. PSG is suggested for suspected sleep-related breathing or movement disorders, while actigraphy may aid in diagnosing circadian rhythm disturbances associated with insomnia.	The statement was removed.
6	According to current evidence, it is recommended to limit drug treatments for insomnia to four weeks due to the rapid development of tolerance. Avoiding dose increases is advised to prevent dependency. Extended treatment periods may be considered in certain cases, with careful consideration of the benefits and drawbacks.	According to current evidence, it is recommended to limit drug treatments for insomnia to two to four weeks due to the rapid development of tolerance. Avoiding dose increases is advised to prevent dependency. Extended treatment periods may be considered in certain cases, with careful consideration of the benefits and drawbacks.
7	While herbal medicines such as valerian, chamomile, and kava are among the most commonly used CAMs for insomnia, there is no statistically significant difference in their efficacy compared with placebo, and their use is discouraged by guidelines.	The statement was removed.
8	LEM is recommended as a first-line treatment for sleep maintenance in insomnia when initiating treatment.	LEM is recommended for sleep maintenance in insomnia when initiating treatment.
9	BZDs should be used with caution in elderly patients with dementia, cognitive impairment, or those prone to falls and fractures, due to their heightened sensitivity to depressants affecting the central nervous system.	BZDs should be avoided in elderly patients with dementia, cognitive impairment, or those prone to falls and fractures, due to their heightened sensitivity to depressants affecting the central nervous system.
10	Zolpidem can be a suitable treatment for insomnia, but prescribing and dosing should be determined on a case-by-case basis, considering the patient’s physical and psychiatric risk factors.	Pharmacological intervention can be a suitable treatment for insomnia, but prescribing and dosing should be determined on a case-by-case basis, considering the patient’s physical and psychiatric risk factors.
11	Licensed drugs, such as BZDs and suvorexant, are effective for the acute treatment of insomnia. However, they often have poor tolerability, and data on their long-term effects are limited.	Many licensed drugs, such as BZDs, LEM, and suvorexant, are effective for the acute treatment of insomnia. However, they often have poor tolerability, and data on their long-term effects are limited.
12	Both zolpidem and zopiclone effectively initiate sleep with a rapid onset. Zolpidem, preferred for short-term use, raises concerns about next-day cognitive impairment in older adults. It is as effective as zopiclone, with fewer rebound effects upon discontinuation and better overall tolerability.	Zolpidem effectively initiates sleep with a rapid onset. Preferred over BZDs for short-term use, it raises concerns about next-day cognitive impairment in older adults.
13	Zopiclone effectively treats insomnia by reducing SL, nocturnal awakenings, and WASO, while increasing TST. However, its dose should be gradually tapered to minimise the risk of rebound insomnia.	The statement was removed.
14	The minimum effective dose of BZDs and Z-class drugs should be used only when essential and for less than four weeks, especially in individuals over 60 years of age.	The minimum effective doses of Z-class drugs should be used only when essential and for less than two weeks, especially in individuals over 60 years of age. All pharmacologic interventions should be combined with nonpharmacological interventions to minimise the duration of drug therapy.
15	Transitioning to LEM from other insomnia medications is highly effective, with 95% of patients successfully switching by the end of titration. Patients reported improved satisfaction based on PGI-I and ISI scores, with mostly mild or moderate TEAEs. LEM provides a well-tolerated and beneficial option for patients dissatisfied with current treatments, potentially reducing or discontinuing BZRA use.	Transitioning to LEM from other insomnia medications is highly effective. Patients reported improved satisfaction based on PGI-I and ISI scores, with mostly mild or moderate TEAEs. LEM provides a well-tolerated and beneficial option for patients dissatisfied with current treatments, potentially reducing or discontinuing BZRA use.
16	Quetiapine, an antipsychotic, is occasionally used off-label as an adjunct therapy for insomnia in patients with other psychiatric conditions (e.g., schizophrenia) but is associated with a significant risk of dependence.	Quetiapine, an antipsychotic, is occasionally used off-label as an adjunct therapy for insomnia in patients with other psychiatric conditions (e.g., schizophrenia), but it is associated with notable clinical side effects.
17	LEM exhibits a low potential for dependence, minimal muscle relaxant effects, limited somnolence, and little impact on cognitive function, suggesting it may be a safe and effective option for treating insomnia in real-world clinical practice.	LEM exhibits a low potential for dependence, minimal muscle relaxant effects, limited somnolence, and little impact on cognitive function.
18	LEM’s efficacy in key sleep parameters (TST, LPS, and SE), along with its safety profile—comparable to other treatments in terms of SAEs and withdrawals due to AEs—makes it a compelling first-line treatment option for primary insomnia. Its consistent safety and effectiveness across all age groups further support its widespread clinical use.	LEM’s efficacy in key sleep parameters (TST, LPS, and SE), along with its safety profile—comparable to other treatments in terms of SAEs and withdrawals due to AEs—makes it a compelling treatment option for primary insomnia. Its consistent safety and effectiveness across all age groups further support its widespread clinical use.
19	LEM demonstrates sustained effectiveness in improving sleep onset and maintenance over twelve months of continuous treatment, with no evidence of rebound insomnia or withdrawal symptoms upon discontinuation, indicating potential long-term benefits for adults with insomnia.	LEM demonstrates sustained effectiveness in improving sleep maintenance over 12 months of continuous treatment, indicating potential long-term benefits for adults with insomnia.

AE = adverse event; BZD = benzodiazepine; BZRA = benzodiazepine receptor agonist; CAM = complementary and alternative medicine; DBAS = dysfunctional beliefs and attitudes about sleep; ESS = Epworth Sleepiness Scale; ISI = Insomnia Severity Index; LEM = lemborexant; LEM5 = lemborexant 5 mg; LPS = latency to persistent sleep; PGI-I = Patient Global Impression – Insomnia; PSG = polysomnography; SAE = serious adverse event; SCI = Sleep Condition Indicator; SE = sleep efficiency; SL = sleep latency; TEAE = treatment-emergent adverse event; TST = total sleep time; WASO = wake-after-sleep onset
